# Human-centered design and early evaluation of an interface for mobile-manipulator-mediated pediatric occupational therapy

**DOI:** 10.3389/frobt.2025.1520216

**Published:** 2025-03-18

**Authors:** Rafael Morales Mayoral, Samuel W. Logan, Naomi T. Fitter

**Affiliations:** ^1^ Collaborative Robotics and Intelligent Systems Institute (CoRIS), Oregon State University, Corvallis, OR, United States; ^2^ Disability and Mobility Do-it-Yourself Co-Op, Oregon State University, Corvallis, OR, United States

**Keywords:** assistive robotics, pediatric occupational therapy, user interface design, play, mischief, mobile manipulators

## Abstract

Assistive mobile robots can play an important role in supporting individuals with disabilities. While the field of robot control interfaces for individuals with disabilities is growing, there is little work done on such systems for children end users specifically. Accordingly, we pursued the design of an adapted robot control interface for use in child pediatric occupational therapy (OT). Our target end user, a nine-year-old child with cerebral palsy, leveraged the interface to perform instrumental activities of daily living (e.g., play) with a modern mobile manipulator. We used an iterative design process to adjust and improve the interface via input from the participant’s caregivers and occupational therapist, as well as objective participant performance data. Furthermore, we tested the participant’s ability to utilize our interface by creating two testing cases: a control case (in which our participant performed standard ALD/IADL tasks) and an experimental case (in which our participant performed ADL/IADL practice activities more tailored toward the child). Key insights during the process included the need for sensitivity to taking up space on the child user’s existing power wheelchair, the advantages of integrating technologies familiar to the child (e.g., gaming controls, iPads) in our system design, and the potential value of integrating playful mischief (including playful interactions between the child, their caregivers, and their clinicians) as a part of the playbook for pediatric OT. This work can serve to inform and augment new OT strategies for the marginalized population of young children with disabilities.

## 1 Introduction

Children’s active play holds significant importance in fostering the development of fundamental motor, cognitive, and social skills (Leonard, 2016). Regrettably, many children with mobility-related disabilities have limited ability to engage actively and playfully with their surroundings (Wrotniak et al., 2006). Reduced motor proficiency often leads to difficulties in performing essential activities of daily living (ADL), such as eating and dressing, and instrumental activities of daily living (IADL), such as tidying up toys (Wentz, 2016). One potential solution to address these challenges is the implementation of robot-assisted ADL and IADL support. However, research in this area has predominantly focused on adult users, with limited attention on children, despite the potential long-term benefits of robot-mediated interventions. Our work aims to fill this gap.

More broadly, the presented work represents a challenge to the current *status quo*, in which children’s rights can be easily overlooked. For example, existing research in the area of robot arm systems predominantly emphasizes solutions for adults (Hu et al., 2017; Huete et al., 2012), perhaps due to the greater federal agency funding available for topics such as assistive older adult care (compared to funding for researching child health) (Mitchell, 2017). Research persists on assistive robot arms for children regardless; for example, past rehabilitation robotics work used pre-recorded robot motions (demonstrated by typically developing children) to physically assist children with motor disabilities via robot-child arm couplings (Cook et al., 1988). Another project studied chARMin, an exoskeleton arm designed to guide and assist child users during shoulder and elbow movement (Keller et al., 2013). Other work allowed children with motor disabilities to activate predetermined actions by a robot arm with switches (Cook et al., 2005). A final example used a social robotic coach to aid children in practicing ADL and IADL by verbally explaining the steps needed to perform an action (López-Sastre et al., 2021). Despite the increasing deployment of assistive robotic technologies for children, particularly those with disabilities, the area of robot-mediated occupational therapy (OT) remains largely overlooked compared to analogous work with older user populations. In particular, it is unusual to find assistive robotics work that involves child end users – and even more uncommonly full sets of children, caregivers, and clinicians – in the human-centered design process.

**FIGURE 1:**
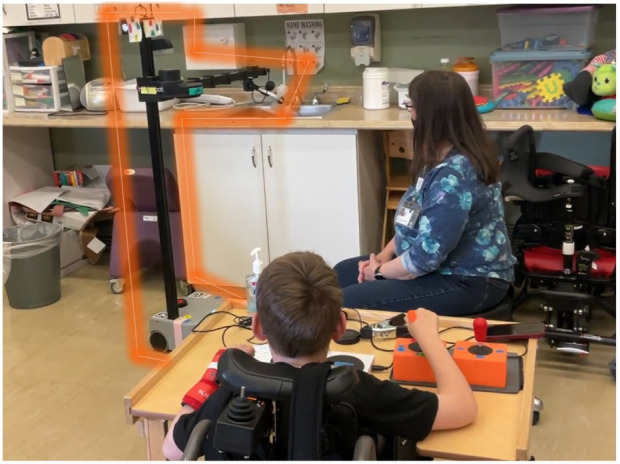
The child participant interacting with one of the interface prototypes, maneuvering the robot to complete the task of filling a water bottle. The robot is surrounded by an orange outline, to highlight its position in the image.

Our first research objective was to *conduct the human-centered design of an intuitive interface tailored for a child user with motor and cognitive disabilities*. This interface is part of a broader robotic system that is meant to facilitate engagement in ADL and IADL via the Stretch RE2 from Hello Robot Inc (Kemp et al., 2022), a mobile manipulator comprising a robot arm mounted on a small-footprint mobile base, as seen in Figure 1. We engaged in a design thinking process in collaboration with a local pediatric rehabilitation clinic, a clinician who worked there, a child end user with cerebral palsy, and the child’s caregivers, as further detailed in Sections 3–5. Our interface prototypes, which varied from tangible buttons and joysticks to fully digital onscreen layouts, were iteratively tested by our end user with the other project stakeholders present.

Our second research objective was to *more carefully evaluate how tailored activities might affect an OT session*. This research goal was developed based on observations during the process of addressing the first objective; the team noticed that the end user seemed to most enthusiastically enjoy performing mischievous activities with the robot (especially in relation to their caregivers and clinician). Follow-up testing methods and results, which more intentionally manipulated mischievous activity as part of the administered OT, are described in Sections 7, 8.

Key contributions of this work include details on our system prototypes, which others may be able to integrate in similar assistive robotic applications; new insights on robot-mediated ADL and IADL for children with disabilities, which expand the field of assistive robots for children; and insight into how tailored activities (like mischievous play) can have a positive impact in robot mediated-OT, which should be considered by researchers when implementing studies involving children. We believe that in a similar way to powered mobility research [e.g., (Feldner et al., 2016; Logan et al., 2014; Logan et al., 2016)] helping to pave the way to new commercial solutions such as the Permobil Explorer Mini, research on pediatric robot-mediated OT can help inform and drive innovation and improved outcomes for children with motor and cognitive disabilities.

## 2 Related work

Below, we present related work on child human rights within the technology sphere, technology systems tailored to aid children with motor and cognitive disabilities, and design thinking in robotics.

### 2.1 Technology and children’s human rights

The issue of children’s rights and agency is often overlooked, and many adults fail to include children in discussions that directly impact them. Outside of the technical realm, custody battles and education offer two common examples of this problem. In the former case, children frequently lack a voice in determining their custody arrangements, despite many expressing a desire to be consulted in the decision-making process (Birnbaum and Saini, 2012). Likewise, within elementary school educational settings, children’s voices are frequently disregarded in decision-making processes. This oversight is problematic because when teachers actively listen to children’s perspectives, it can enhance those students’ confidence in their skills and capabilities (Sirkko et al., 2019). Moreover, incorporating children’s input into activity selection can foster playful learning environments, making learning more enjoyable for students and promoting better outcomes (Baker et al., 2023).

The inclusion of children’s voices in the design of technology follows a similar pattern. For example, mainstream companies have largely overlooked assistive clothing for children (Javed, 2021). On a smaller scale, advocates for change (e.g., Chelsea Funk from the Columbus College of Art and Design) are committed to assisting children with disabilities by crafting comfortable assistive clothing customized to individual needs (Columbus College of Art and Design, 2018). A current missed opportunity is pursuing this type of work on a broader scale. In assistive robotics work, efforts focused on ADL and IADL, tend to overlook the preferences and unique requirements of children, instead focusing on adult (and especially older adult) demographics. As one specific example, in the realm of assistive upper body exoskeletons, the predominant focus has been on adults, resulting in limited availability of systems tailored towards children (Babik et al., 2016; Rahman et al., 2006). This adult-centric design has led to exoskeletons that are bulky and poorly adapted to children’s smaller bodies and unique movement patterns, making them unsuitable for supporting a child’s everyday needs. It was not until the development of the “Playskin Lift” in 2014 (approximately 54 years after the introduction of the first exoskeleton system) that the first garment exoskeleton system was designed for a younger demographic, with updated features such as lightweightness and compactness (Hall and Lobo, 2018). The needs of both groups are important, but focusing on the needs of adults rather than children leads to essential missed opportunities in supporting early development, which subsequently leads to better outcomes throughout all of life (Anderson et al., 2003; Black et al., 2017). Accordingly, the presented work focuses on human-centered design with a child end user and their care network closely in the loop. We encourage more consideration of the needs of children in assistive robotics research generally, as well as close engagement with children in any technology intended for young end users.

### 2.2 Existing assistive technology for ADL/IADL support

A wide range of motor rehabilitation systems can help individuals with disabilities to successfully carry out tasks related to ADL and IADL. For example, exoskeletons can support motor function related to carrying out daily tasks (Yamamoto et al., 2002; Farris et al., 2011). Additionally, body weight support systems like gait trainers (Shih et al., 2023; Mehrholz et al., 2017b; Morone et al., 2017; Hesse et al., 2012) and harness systems (Helmi et al., 2023; Mao et al., 2015; Mehrholz et al., 2017a) enable individuals of varying ages to practice walking and lower extremity movement. Wheelchair-mounted robotic arms allow users to independently interact with more of their environments than would otherwise be possible (Maheu et al., 2011; Routhier et al., 2014). These systems can be used in rehabilitation, but factors such as size, weight, and location of use constraints often limit their usage in OT settings specifically (for example, by interfering with interaction with objects or other elements of the user’s surroundings, as is often needed in OT). Specifically, most exoskeletons are bulky and require individuals to have typical cognitive development for effective use. Gait trainers and harness systems often are confined to a specific location due to their size and weight. Wheelchair-mounted robotic arms have a limited range of reach. Our implementation of robot-assisted ADL and IADL aims to tackle some of the main limiting factors listed above (e.g., size, location, and reach restrictions).

In addition to systems for motor rehabilitation, past work has considered assistive technologies for OT specifically. Many of these systems are focused on supporting ADL for older end users (adults, which especially much work focused on older adults), but their example can still guide and inform the current work with child users. One example is the Activities of Daily Living Exercise Robot (ADLER), a robotic arm system designed to physically support a user’s arm and aid in reaching and grasping tasks necessary for ADL (e.g., picking up food) (Johnson et al., 2006). Exoskeletal robotic systems like those mentioned above have also been integrated into conventional OT to enhance activities of daily living for adults affected by stroke (Iwamoto et al., 2019). Additionally, custom mobile-based teleoperated robots have been implemented to assist the elderly with dementia in performing tasks like making a cup of tea (Begum et al., 2013). Most closely to our presented work in the OT space, past research with a Stretch robot used the system to assist an older adult in performing ADL (e.g., self-feeding) and IADL (e.g., delivering a rose to his spouse) using a customized interface (Nguyen, 2021). Although robots have been integrated with selected OT tasks for older adults, similar research with a younger population has lagged behind. In our work, we seek to support this segment of the population that can greatly benefit from enhanced ADL/IADL abilities.

### 2.3 Design thinking for assistive robots

Design thinking is an iterative human-centered design process that focuses on authentically understanding end user experiences/goals/values, developing design ideas, and implementing and testing a range of prototypes (Razzouk and Shute, 2012). Accordingly, design thinking is typically segmented into five distinct but interlocking phases: empathize, define, ideate, prototype, and test. Design thinking is a fitting process choice in robotics when there is no predefined solution and a candidate system needs continuous refinement to better align with user requirements. (User-centered design is another option that can support a successful design process; however, this approach focuses on digital user interfaces specifically (Kraft, 2012).) One example of design thinking in the socially assistive robotics space was the design process of a robot designed to engage in conversation with people with Alzheimer’s disease (Mitchell et al., 2021). The design thinking process for Stevie, an assistive robot for skilled nursing facility settings, involved understanding needs of the intended deployment space, iterating through multiple prototypes, and assessing each candidate solution based on end user feedback and fly-on-the-wall observations (McGinn et al., 2020). Our work builds on these successful past examples of design thinking in a new application domain; although many of our prototypes ended up centering on user interface iterations, we did not know that this would be so when beginning the work, and we selected a more general-purpose design process.

## 3 Empathize and define phases

Design thinking begins with a deep dive into empathy, prioritizing an understanding of the circumstances of the intended end users and their networks of caregivers. This empathizing process involves actively seeking information about current situations, desires, and requirements. In our case, we saw the successful past example of Stretch robot-mediated OT in work mentioned above [i.e., (Nguyen, 2021)] and wondered if a similar beneficial intersection might exist between modern mobile manipulator technology and the OT of young children with motor and cognitive disabilities. Leveraging an existing relationship with the Oregon Health and Science University (OHSU) Doernbencher Eugene Child Development and Rehabilitation Center (CDRC) [which led to publications on early childhood physical therapy (Helmi et al., 2022; Helmi et al., 2023)], we contacted their OT services to perform a semi-structured interview with an occupational therapist and conduct onsite observations.

Our early phone-based interview with the occupational therapist lasted 1 hour. During this call, we discussed potential interest in Stretch robot-mediated OT and children who might be a good candidate for this type of interaction. A prospective end user would need to have the ability (both physical and cognitive) to control the robot and be able to augment motor abilities with the introduction of the robot. We also learned more about typical ADL and IADL practice in pediatric OT interactions; for example, the IADL of play is a common central theme of therapy sessions.

During our visit to the CDRC’s OT services, we met several children engaged in occupational therapy, including one child (a nonverbal 9-year-old male diagnosed with cerebral palsy with motor and cognitive impairment) who navigates using a power wheelchair and seemed to be a good candidate for mobile robot-mediated OT. Despite extremely low hand mobility in the left hand (i.e., only being able to lift the arm to a chest level, and not being capable of arm extension or grasping without moderate assistance), and partial movement in the right hand (i.e., being able to lift the arm to a face level, nearly being able to perform a full hand extension, and being able to grasp small objects such as crayons), this child is able to use joystick-based control of a power wheelchair. However, his range of motion poses challenges for interacting with objects at varying heights in the environment, such as toys and water cups for IADL and ADL, respectively, which the OT would otherwise be interested in using in his therapy sessions.

To gather more information about this child specifically, we collected field notes at a subsequent scheduled therapy session. During this session, our team conversed with the caregivers and discussed topics such as the child’s personality traits and the potential benefits of the robot for supporting child ADL/IADL goals. From this information, we gathered that the child was somewhat mischievous and enjoyed playfully pranking the people around him. Potential robot-mediated OT benefits included enabling the child to perform ADL-related tasks, such as fetching water independently, and participate in a broader range of developmentally crucial activities, including play–a fundamental IADL in pediatric OT that fosters cognitive, social, and motor skill development. The occupational therapist confirmed that these types of interactions were part of the child’s current OT goals, and also highlighted past experiences that the prospective end user seemed to enjoy. This list included the child interacting with others (e.g., the caregivers and therapist) and engaging in playful mischief with the people around him (e.g., deliberately typing incorrect words when working with a text-to-speech device and aiming a ball-launching robot at their caregiver to spur a playful “chase” scenario). (Although we did not include mischief in the distilled design requirements below, we circle back to the idea of mischief in the design of the research efforts discussed in Section 7).

Based on the interview and observations, we considered the potential of introducing a Stretch-based system to the identified child’s OT sessions to serve his needs and interests. In collaboration with the occupational therapist, our team, we brainstormed system requirements to ensure the system’s usefulness and usability in such a scenario. This process led to four essential requirements for the system, as outlined below.

• Approachable: The system needs to be easy to use. This might include (for example,) a simple and minimalist system with large control elements, since the user’s condition causes small tremors that would make it difficult to use small buttons/controls. The system should also feel natural to use, likely by mirroring the design of other systems the user is familiar with.
•Easy to set up: The system must be possible to set up and removed in as few steps as possible to make it accessible to care partners, OTs, and other clinicians.
•Compact: Due to small open space footprints in the deployment areas and the limited motion of the participant, the system’s footprint must be compact while in use. The system also must be portable enough to be carried, for easy transportation in and out of prospective deployment areas such as the clinic and home.
•Useful for tidying up, self-feeding, and playing: The therapist and caregivers highlighted the need for the robot to support selected ADL/IADL that were appropriate for the end user. In particular, we considered the ability to play (or “be a kid”) to be important, since it is often underserved but can enhance cognitive and motor development (Lai et al., 2018).

## 4 Ideate

During the ideation stage of design thinking, the objective is to generate a range of potential solutions to address the issues identified in the earlier stages. Based on the requirements of compactness and portability, the Stretch robot appeared to be a viable system for enabling new child interactions with the environment. The Stretch robot also seemed to have good potential for easy setup, as long as any control interface hardware was not unduly obtrusive. This left most of the ideation questions for the application of interest in the domains of allowing the child to easily control the robot and adapting activities to be performed with the support of the robot.

At a high-level, the Stretch robot offers certain advantages over a wheelchair-mounted robotic arm since relative to the footprint size of a wheelchair, the Stretch base is smaller and can reach extra areas of the surroundings. The robot can also reach the ground (differently from most wheelchair-mounted arms). Another unique characteristic of Stretch is the simpler degrees of freedom compared to many robot arms; the two degrees of freedom of the arm (one up/down, one telescoping in/out) can lead to easier control for the participant. In designing the interface for the robot, we sought to treat the robot as an extension of the user (rather than a fully autonomous entity or something controlled by the caregiver). This approach prioritizes the participant’s independence, enabling them to control the robot, rather than relying on a robotic system to act as a caregiver. Further, the process of teleoperating the robot can support fine and gross motor skills (via movement of the hands and other body parts, such as the head or torso/core, to use the interface) and cognitive development (via thinking about what steps to take in sequence).

**FIGURE 2:**
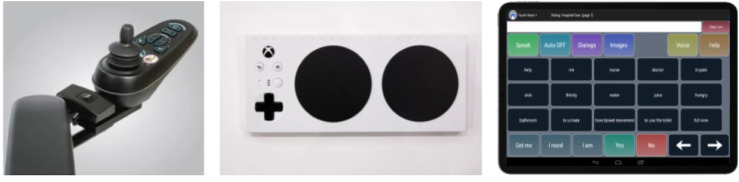
Interface options from our ideation that guided later prototyping. Left: controls of a power wheelchair. Middle: adaptive Xbox controller. Right: tablet-style device like the one that the child uses for homework.

Prior to exploring new interfaces for controlling the robot, the team experimented briefly with the out-of-the-box supported method for teleoperating the robot: an Xbox-style gamepad. The end user had prior experience using the joysticks of an Xbox controller, so we considered that this solution could be a useful benchmark in the design process and potentially provide us with additional helpful design criteria. While this solution worked as a proof of concept (i.e., the child was able to control the base movement of the robot with the joysticks), the participant found it difficult to press the small Xbox-style gamepad buttons to operate other features of Stretch. This observation motivated us to try other joystick and button-based control, as well as considering hardware interfaces that the child was already familiar with. This line of thinking led us to consider design inspiration from additional devices: current joysticks on the end user’s power wheelchair, adaptive versions of the Xbox controller with more usable buttons, and tablets (which were familiar to the child from other applications, such as engaging in daily homework and using text-to-speech applications for practicing sentence construction). These interface options are shown in Figure 2.

During this design stage, we also sought to connect broader ADL and IADL categories of interest with more specific action ideas that could fit well with Stretch’s abilities and measurable outcomes. The broader set of activities considered by the research team included: stacking wooden blocks (IADL of play), picking up small buckets (IADL of play), playing custom games of bean bag toss (IADL of play), filling up a plastic bottle with water (for the ADL of drinking), collecting items from individuals around the room (IADL of play), and delivering items from one point to another (for the ADL of functional mobility). We pared this list down to the three ultimate ADL-relevant tasks chosen by the therapist and caregivers for evaluation: grasping an item at floor level, retrieving an item from atop a table, and removing an item from inside of a cabinet.

## 5 Prototype/test

The next design thinking steps were to create and test prototypes based on the ideas from the previous phase. We developed four candidate interfaces for controlling Stretch, and we iteratively evaluated each option using consistent methods. Since the evaluation methods were the same for each prototype, we first introduce the methods in Section 5.1 before outlining each prototype and the results of their evaluations in the following subsections.

### 5.1 Methods for testing prototypes

Each prototype discussed below was tested using the same procedure to allow for easy comparison and continual improvement.

#### 5.1.1 Interaction premise

**FIGURE 3:**
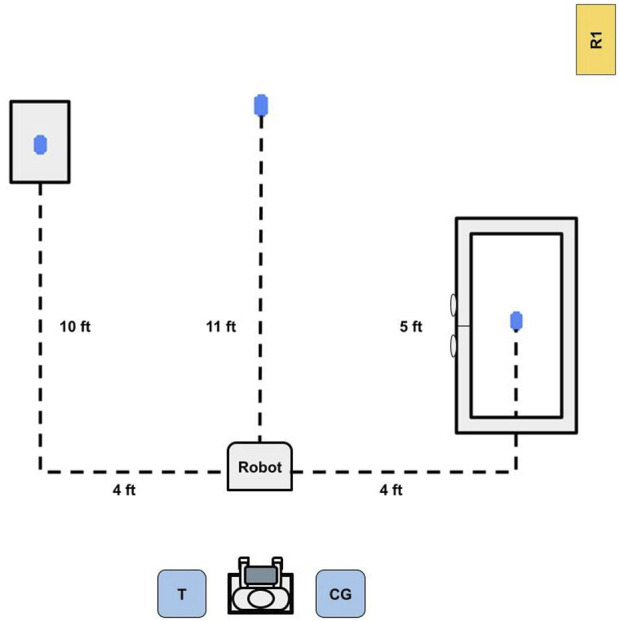
Top-down layout sketch of the testing environment. The user operated the robot with guidance from the therapist (denoted as T) to pick up three objects, represented by the blue ovals; one on the floor, one on a table, and one within a closed cabinet. The caregiver (denoted as CG) was also present for support and reassurance throughout the process, and the researcher (denoted as R1) was present to record field notes.

The mock ADL- and IADL-relevant tasks used in the evaluation were selected based on the target premises identified in the ideate stage. An illustration of the evaluation setting appears in Figure 3. Within this space, the three tasks used for evaluation were:

•Collecting an item from the floor: This task aimed to fill the participant’s currently unfulfilled need to reach the ground to be able to pick up items, for example, to retrieve an item that falls or to pick up after oneself.
•Retrieving an item from atop a table: This task sought to fulfill the common need to pick up items at hip level (about 2.8 ft or 0.85 m off the ground). Achieving this is helpful for retrieving items to do common ADL and IADL tasks, such as manipulating water bottles or toys.
•Removing an item from inside of a cabinet: This task was intended to support the manipulation of objects in multi-step interactions, which may involve more complicated perception situations such as occlusion. Again, this type of ability can support object manipulation and retrieval scenarios related to ADL and IADL (i.e., collecting a more obscured object in order to next do something with it, such as removing a glass from a cabinet before filling it and bringing it to a table).

If time allowed, the participant additionally had the chance to try different activities with the robot based on the therapist’s recommendation.

#### 5.1.2 Procedure

The testing occurred during 30–45 min sessions during which the participant used the Stretch teleoperation interface to collect three items via the tasks listed above. During each session, the participant was led to a designated location in the space with a good vantage point for the coming tasks. The researchers placed three objects (i.e., a bean bag, a cup, and a small toy frog) in the environment (as visualized in Figure 3). The participant was asked to use the prototype interface to teleoperate the robot and collect each object and bring it to the region around himself and the care team. The therapist and caregivers were able to give the participant hints and direct physical help as needed but were asked to minimize the physical interaction they had with the prototype. Both the therapist and the caregivers also refrained from physically interacting with the robot. The order of the first two tasks (i.e., fetching an item from the ground and a table) was balanced, but removing an item from inside of a cabinet was always evaluated last because it required more time. We made this decision to ensure a consistent ability to compare the first two tasks even if the third could not be fully completed within a session. The participant’s family was not compensated for these early prototyping sessions.

#### 5.1.3 Measurement

We recorded field notes live during each session, in addition to gathering third-person camera footage from the view of the participant during the sessions. After each session, we completed and recorded a semi-structured interview with the participants’ therapist and caregivers. We also asked the occupational therapist to complete two surveys based on common product design scales (Cheng, 2024):

•The Customer Effort Score (CES) scale, which captures the level of effort required from the user on a 7-pt scale from “Very Easy” (1) to “Very Difficult” (7)
•The Customer Satisfaction Score (CSAT) scale, which centers on user satisfaction with the system on a 7-pt scale from “Very Unsatisfied” (1) to “Very Satisfied” (7)

Overall, this subjective data was collected from the therapist and caregivers since the child was unable to directly self-report this information. Compared to the research team, the clinician and family had established strong rapport with the participant to better be able to gauge the child’s responses.

#### 5.1.4 Analysis

We used anecdotes from the field notes and interviews, in addition to raw survey data, to reason about what may be working well and what may need improvement in each design and inform the next design cycle.

### 5.2 Prototype 1

Based on the ideation phase outcomes, we centered the first major prototype effort around the Xbox adaptive controller (Microsoft, 2024) and related Logitech adaptive buttons (Logitech, 2019) (as designed for easy interfacing with the Xbox adaptive controller and range of button options). Utilizing the adaptive buttons allowed the team to rapidly arrange and rearrange the teleoperation interface setup to fit the needs of the end user. The commercial joysticks offered with the adaptive controller proved insufficient for our needs (they proved to offer just button input, rather than analog input in multiple directions), so we developed two custom joysticks based on reverse-engineered arcade controls.

**FIGURE 4:**
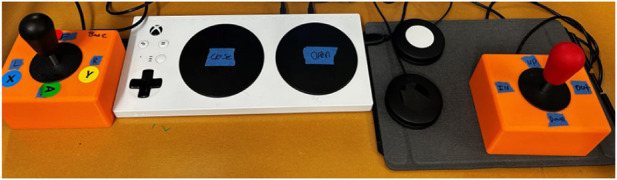
Image of interface prototype 1, which consisted of an Xbox adaptive controller, adaptive buttons, and two joysticks.

The resulting interface prototype, as shown in Figure 4, featured physical buttons and joysticks to allow for the participant’s control of the robot’s motions, with one joystick allowing control of base movement (i.e., linear and angular) and the other controlling the arm movement (i.e., lower, raise, expand, and contract). The Logitech adaptive buttons controlled the yaw movement of the gripper, with one button controlling the left yaw and another controlling the right yaw. The large buttons in the center of the adaptive controller opened and closed the gripper. The system also had two arcade-like joysticks, where one joystick controlled the base movement and the other joystick controlled the arm movement of the robot.

#### 5.2.1 Testing results

The prototype, which was tested in two sessions, had promising results. For example, the occupational therapist stated that *“this system could really help children with impaired movement perform tasks that they would not be able to otherwise.”* The occupational therapist also expressed their thoughts on how engaging the robot could be for child users, mentioning that *“it’s fun and motivating for the kids, which makes it a great tool to use as a therapist! I really appreciate the versatility.”* The main problem that arose from this system was how difficult it was for the participant to remember the mapping of buttons/joysticks to robot functions as well as how unintuitive the mapping was for our end user. The occupational therapist mentioned that *“[the robot needed improved] responsiveness and sometimes [it was] not as intuitive to use. [It] requires good motor control.”* On the CES and CSAT scales, the interface received a score of five by the therapist when it came to the amount of effort needed to use the system and a score of two in satisfaction with the device. She stated that *“the joysticks and switch setup [buttons] required the participant to remain at a table and did not allow for any mobility”* when discussing one major area for improvement; this prototype involved so many distinct hardware pieces that although designed to sit on the tray of the end user’s wheelchair, use of the interface in the end required placement on a separate table.

### 5.3 Prototype 2

In the previous prototype iteration, main pain points involved the intuitiveness of the control interface and the amount of hardware associated with the system. Our next prototype (prototype 2) specifically sought to improve the intuitiveness aspect. For this prototype, the team implemented a display in the form of an iPad connected to one of the robot cameras. The fisheye-style camera was located on the robot’s end effector and transmitted footage to the user via an onscreen Virtual Network Computing (VNC) viewer display. Although the robot was used in the same room as the participant, adding a camera made it possible to detect items near the gripper that may have been visually occluded from the user’s perspective. This update also allowed the end user to have a better conceptualization of the robot’s position (as they themselves could not change positions to gather different views of the robot progress and orientation in the room).

**FIGURE 5:**
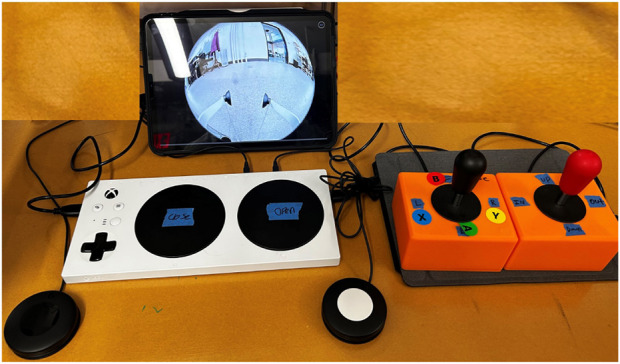
Image of prototype 2, which consisted of an Xbox adaptive controller, assistive buttons, and two joysticks to control the movement and actions of the robot, as well as a iPad-displayed camera view of the environment from a fisheye camera located in the robot’s end-effector.

The prototype, as seen in Figure 5, provided a fisheye first-person view from the perspective of the robot gripper, to support easier navigation of the environment with a gripper camera view that was programmed to always match the orientation of the robot base. Utilizing the gripper camera allowed the end user to view the environment and detect objects, as well as being able to more easily detect if they had a successful grasp. The ability for the end user to move the robot arm and gripper while maintaining the camera view on the gripper was intended to allow for more complex movements. The first-person view allowed the end user to bypass needing to keep track of the robot’s orientation; rather, the joystick controls always matched the direction the camera view was displaying.

#### 5.3.1 Testing results

During the testing phase of prototype 2, which spanned three sessions, the added display notably improved the user’s ability to navigate their environment. This observation was also acknowledged by the therapist. Due to having a perspective of where the gripper was aiming, the participant could position the gripper in a way that allowed for more fluent grasping without needing to reposition the robot arm and gripper multiple times before successfully grasping an object. The display also made it possible for the participant to grasp items that were not directly in sight from the user’s position in the experiment setup. With this iteration, the participant was able to grasp items such as cups that were located in a different part of the deployment area and outside of the view of the participant. The added display enabled the participants to use the camera to check for obstacles in front of the robot, making it easier to maneuver. For example, they could use the camera to see if there was an object in front of the robot that would inhibit its movement. Adding video feedback appeared to increase the levels of satisfaction and performance of the child; the caregiver stated that the end user *“picked [objects] up so much faster than he did last time”* (i.e., faster than during sessions with prototype 1) when discussing the performance of the child. Even the child gave direct satisfied feedback when the therapist asked *“is this a ‘winner-winner chicken dinner?”’* and the child responded by smiling and making nonverbal sounds expressing happiness. While the updates refined the system to a more usable state, it still included the portability challenges of the previous prototype. For example, the therapist noted that *“the joysticks and switch set up required the participant to remain at a table and did not allow for any mobility.”* Using the CES and CSAT, the therapist gave this interface a score of four for the amount of effort needed to operate the interface and a three for satisfaction with the interface.

While evaluating prototype 2, we also noticed inklings of playful mischief while using the prototype. During the end of one of the prototype 2 testing sessions, the child was provided an option to play freely with the robot. The therapist suggested that stacking a cup on top of a paper box would be a good exercise. During the stacking process, while the therapist was given instructions, the end user purposely and repeatedly pushed the cup off of the table. This behavior appeared to generate a reaction from the therapist; she played along with overreacting, which led to laughing noises from the end user.

### 5.4 Prototype 3

The second lingering need from prototype 1 was a more minimal hardware footprint. Although we had at first intuited large controls and physical hardware to offer accessibility advantages, after talks with the therapist, we decided to try an interface design that centered on touch interactions with an iPad, a familiar system to the end user that he uses for activities such as completing homework. The interface was designed using PYQT five and utilized Robot Operation System (ROS) to communicate between PYQT and the robotic system. During this change, the team decided to implement both cameras that were available on the robot. This allowed the end user to have a dedicated gripper view (which helped with manipulation) as well as a camera view of the environment (as seen in most telepresence robot systems). Previous physical button functionality was replaced by click input functionality with the camera footage being displayed on the interface.

**FIGURE 6:**
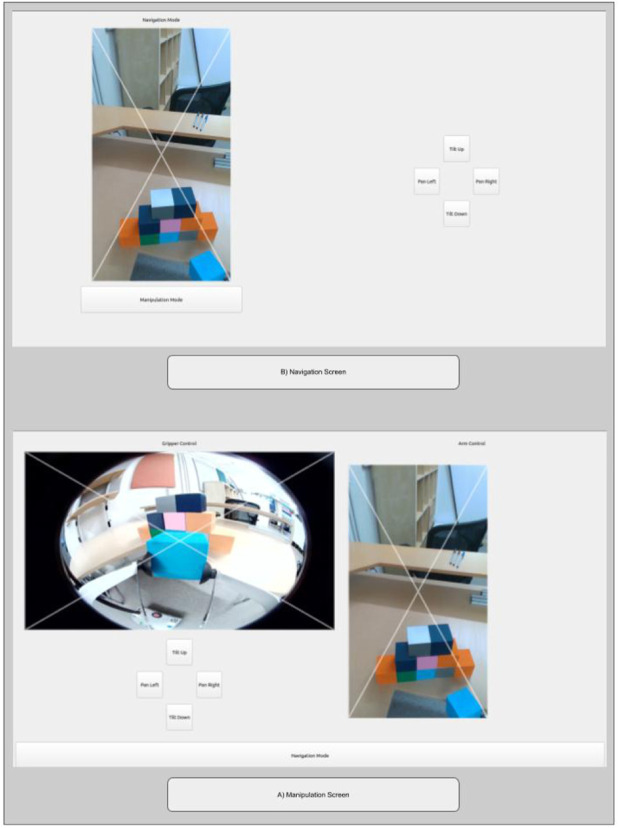
Prototype 3, which consisted of two screen options between which the user could toggle. The navigation mode (top screen) allowed the user to control of base movement, and the manipulation mode (bottom screen) allowed control of both the robot arm and the robot gripper movement.

**FIGURE 7:**
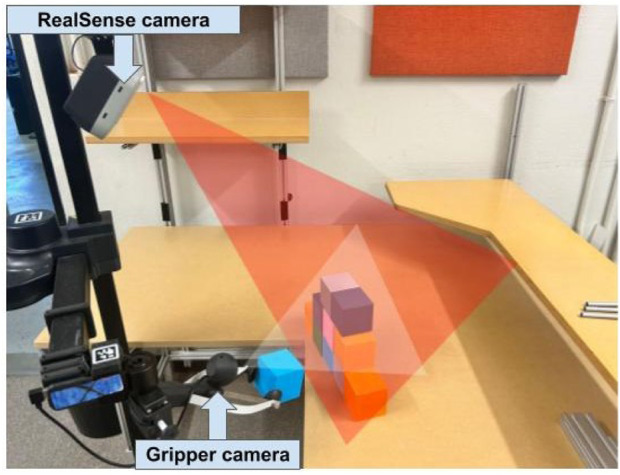
An illustration of how prototype 3 captured video footage using two cameras. The red triangle indicates the perspective captured by the RealSense camera (the rectangular views in Figure 6), and the white triangle illustrates the perspective captured by the gripper camera (the fisheye view in Figure 6).

The prototype, as shown in Figure 6, consisted of two screens that the user could switch between using an onscreen button. The first screen, navigation, consisted of a section that allowed the user to control the movement of the robotic base (i.e., linear and angular) utilizing the RealSense camera stream, and the second screen, manipulation, had two sections, one of which controlled the robot’s arm movements (i.e., lower, raise, expand, and contract) and included the RealSense camera footage and the other of which controlled the gripper movements (i.e., yaw left/right, open, and close) and included footage from the gripper camera. The user could make decisions on how to control the robot using footage of the environment around the robot from the robot’s onboard cameras, as also shown in the interface. An illustration of each camera’s location and field of view appears in Figure 7. Further, the camera footage was set up as four clickable sections that allowed PYQT-based control of the robot. This was done by superimposing an ‘x’ shape on each camera view, which divided the camera view into four triangular sections (i.e., top, bottom, left, and right). When clicks were detected within the four triangles, the interface would prompt a set behavior of the robot, as further detailed in the following few sentences. For the navigation mode, clicking the left or right triangular region of the video feed controlled the angular base movement, while clicking the top or bottom sections controlled linear base movement. For the manipulation mode, clicking on the top or bottom section of the gripper camera view opened or closed the gripper, while clicking the left or right section controlled the gripper yaw movement. Clicking on the top or bottom section on the RealSense camera view (while in manipulation mode) controlled the raising or lowering of the arm, while the left or right section controlled the expanding or contracting of the telescoping second link of the robot arm. Finally, both screens included digital buttons with labels to control the RealSense camera movement. With the button-based input, the end user could scan the environment as needed using the tilt-up and tilt-down buttons to control the pitch movement and pan-left and pan-right buttons to control the yaw movement.

#### 5.4.1 Testing results

During the testing of prototype 3, as conducted in a single session, we observed that consolidating all control inputs within a small area seemed to enhance the end user’s interaction with the system. This layout eliminated the necessity to reach across the desk to access the buttons, which appeared to enhance usability. The interface prototype also made it easier for the end user to visualize the orientation of the robot, since one camera view always faced the forward orientation of the base. Overall, adding different video footage of the robot’s position within an iPad display and implementing the controls in the display itself seemed to increase levels of satisfaction; the therapist stated that *“it was like night and day”* with how comfortable the participant was moving when comparing the prototype 3 session to earlier sessions. These updates also tended to increase the performance of the participant, with the resulting robot movements being more fluent. While the overall system performed better, the research team and therapist noticed a few flaws in the system layout, such as challenged detecting items that were close to the robot base (due to the position of the camera) and the inability to rapidly switch between moving the arm and base movement (due to the prototype’s two-screen setup, which led to some levels of frustration and confusion when the participant forgot which screen they were in and moved incorrectly). The lack of large and descriptive button labels also amplified the confusion towards the end user. When asked about areas for improvement, the therapist stated *“I think just that it was challenging to think about having to switch screens to switch modes [navigation and arm movement], mentally it was easier to keep moving but they needed to make a change between base and arm movement. So, I would say there was a disconnect there.”* Using the CES and CSAT, the therapist gave this interface prototype a score of four for the amount of effort needed to operate the interface and three for satisfaction with the interface.

### 5.5 Prototype 4

After evaluating the last prototype, the team decided to switch the feed from the RealSense front-facing camera out with video from a smaller HD camera that could be attached to a lower point on the robot, allowing the end user to more easily detect objects that were near the robot. With feedback from the therapist, the team redesigned the interface layout to contain all robot movements in one single screen. Adjusting the layout to contain all robot controls allowed for a faster transition between base and arm movement, to alleviate the frustration of switching that the end user experienced with the previous prototype.

**FIGURE 8:**
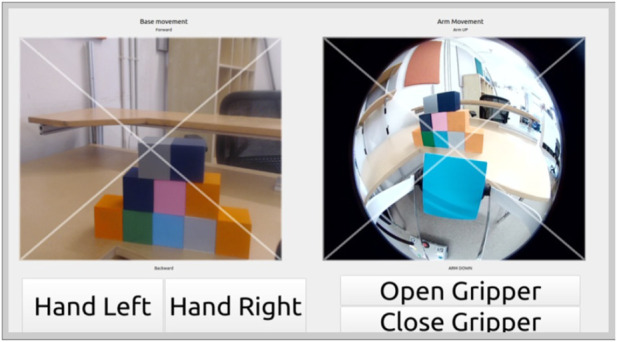
Image of prototype 4, which included one single screen view with base movement (left) and arm movement (right). The gripper was controlled using the four large buttons positioned in the bottom portion of the interface.

**FIGURE 9:**
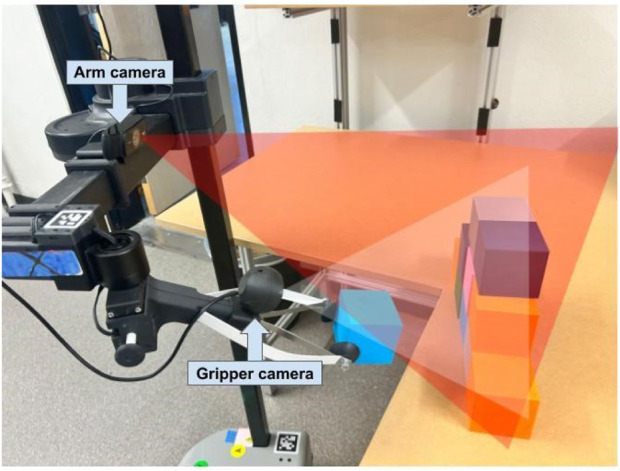
An illustration of how prototype 4 captured video footage using two cameras. The red triangle indicates the perspective captured by the arm camera (forearm-mounted webcam), and the white triangle illustrates the perspective captured by the gripper camera.

The updated prototype, shown in Figure 8, exchanged the previously smaller onscreen gripper controls for large digital buttons with large bold labels describing the function of each button (i.e., hand left and right controlled the yaw left/right, and open and close gripper controlled the gripper state). The interface screen maintained the same method of superimposing an ‘x’ shape on each camera view, which divided the camera view into four triangular sections (i.e., top, bottom, left, and right). The camera feed on the left side allowed for linear and angular movement of the base via the same convention of the similar feed in the prototype 3 navigation mode. The camera feed on the right side allowed for the control of the arm movement (i.e., lower, raise, expand, and contract) using the same convention as the similar feed in the prototype 3 manipulation mode. The arm camera (front-facing camera) was located on top of the robot arm, as seen in Figure 9 which allowed the end user to move the viewpoint of the arm camera by lowering or lifting the robotic arm (akin to switching from walking with your head fully elevated to crouching down for a direct-sightline view of an area of interest).

#### 5.5.1 Testing results

During the testing of prototype 4, which was conducted in two sessions, the rearranging of the interface elements to all fit on one screen allowed the end user to be able to move the robot around and make small adjustments at a faster rate. It additionally removed the frustration (and potentially cognitive overhead) of having to switch between screens. The therapist confirmed this improvement, stating that *“having all the controls on the tablet [at once] is great.”* The caregiver also echoed how fast the end user finished tasks with the new interface, stating *“that was way faster. It was so fast!”* The therapist additionally mentioned that the position of the gripper buttons seemed to make it easier to understand the updated layout. Using the CES and CSAT, the therapist gave this interface a score of two for the amount of effort needed to operate the interface and a six for satisfaction with the interface.

During the final testing session, the team noticed that the end user desired to perform tasks that were more fun and less structured. For example, instead of grasping the item that was suggested to them by the therapist, the participant would direct the robot toward toys in the environment or specific toys that were brought by the participant’s caregiver (i.e., seeking playful interactions). Therefore, in the following session (which were not considered when answering the CES and CSAT), the therapist decided to change the approach of getting the objects, with the therapist creating mock scenarios that leaned more towards playing. Play-oriented tasks appeared to lead to the child enjoying the experience more. Or in other words, retrieving objects as a form of play was more enjoyable than retrieving objects in a structured way.

## 6 Interface discussion

**FIGURE 10:**
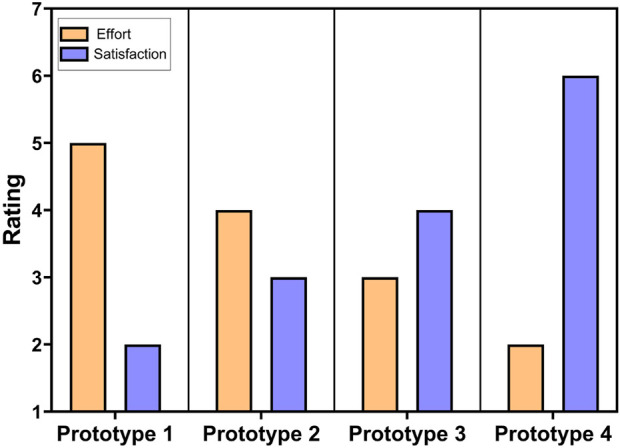
CES and CSAT scores for the prototype-wise perceived effort level needed for the child to use the teleoperated system and how satisfied the therapist was with the system.

Overall, the iterative design efforts presented in this paper continually improved the ease and satisfaction of interface use for our child end user, as shown in Figure 10, and confirmed selected best practices from the assistive robotics literature. Our early prototypes with multiple physical controls were overstimulating for the end user. This finding makes sense since the potential for complex control systems to overwhelm end users is known (Chen et al., 2007), and this trend can be particularly salient for children with cognitive disabilities. Our later and more compact single-screen interface prototypes led to better overall performance by the end user.

Camera perspectives were important for allowing robot adjustments that were faster, apparently easier, and even increasingly complicated. For example, the addition of the first camera view in prototype 2 led to the end user trying simultaneously vertical and horizontal movement control of the robot, a combination that led to more seemingly natural output behavior of the robot. In contrast, adding controls to adjust the RealSense camera complicated the control experience. This insight agrees with selected findings from the field of teleoperation, which showed that adding the ability to control cameras and robot movement simultaneously reduces efficiency due to its complexity (Zhu et al., 2011). As previous studies have shown (Almeida et al., 2020), the misalignments between the frame of reference for vision and action can lead to a higher mental workload. We made a similar observation in work when the participant seemed disoriented by the robot movement in earlier prototypes (i.e., prototypes 1 and 2) before improving this element of the design in prototypes 3 and 4.

Generally, the level of success our end user experienced with the prototypes signals that young children with physical and cognitive disabilities can potentially use systems like the Stretch (or others even more adapted for child users) in pediatric OT experiences. Further, our prototype observations revealed that the end user seemed to want to perform playful mischief using the robot, and the level of focus on the task appeared to be greater when attempting mischief. We therefore propose play (including the potential for mischief) and fun as elements of pediatric OT experience that should be more at the forefront of research and practice; assessments of common topics such as accuracy, metabolism, and efficiency stand to be augmented by considering emotional experience more deeply. This assertion led to one final evaluation in our presented work, which sought to more precisely articulate the potential benefits of tailored OT activities (such as mischief, in the case of the current end user) in pediatric OT.

## 7 Follow-up evaluation methods

Based on the maturing ideas about the interface design and the idea of playful mischief as a part of robot-mediated pediatric OT, we sought to conduct a slightly more in-depth evaluation. We conducted a four-session evaluation with a consistent interface and the same end user as engaged throughout the design process. With this evaluation, we wanted to investigate the potential of tailored activities (i.e., play including opportunities for mischief, as opposed to more standard activities) on robot-mediated pediatric OT. As we prepared for the evaluation, we made minor adjustments to the robot control interface based on a final round of therapist feedback. The final interface and the evaluation design are further detailed in the following subsections.

### 7.1 System details

**FIGURE 11:**
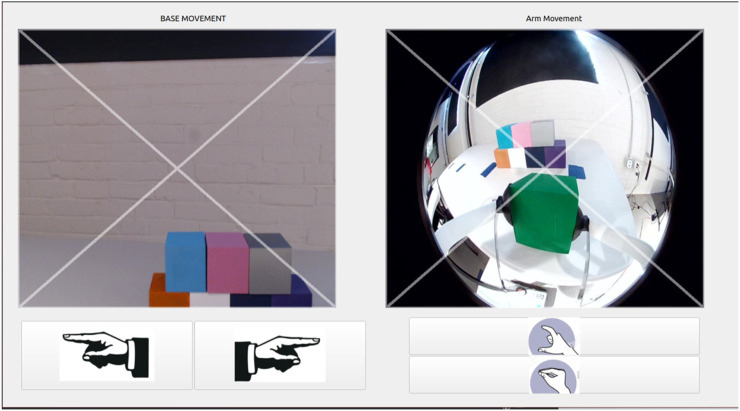
Image of the updated interface, which included one single screen view with base movement (left) and arm movement (right). The gripper was controlled using the four large buttons containing easy-to-interpret images of their purposes.

The robotic system used for this evaluation, as before, was a Hello Robot Stretch RE2. For the current effort, the robot was controlled by a minorly updated version of the prototype 4 interface. Specifically, a first update was that in the new version, a verbal description of the action being performed by the robot (e.g., arm left, arm right, open, close), played from the robot’s onboard speaker. To prevent excessive repetition of the audio, a 25-click minimum limit was set before each audio cue was replayed. The second update was replacing the text labels of the digital buttons with images matching the action that the button would spur (e.g., “close gripper” was represented by a hand closing). These updates were suggested by the therapist, who noted that the participant was sometimes unsure of whether a button press yielded the desired action from the robot and occasionally had difficulty reading the text of the buttons. Figure 11 shows the updated interface.

### 7.2 Evaluation design

To examine the effect tailored activities have on robot-mediated pediatric OT for ADL/IADL support (compared with more usual ADL and IADL activities), we conducted a single-user evaluation that spanned four sessions with two item retrieval tasks each.

We considered two conditions:

• A *control* condition in which the participant performed the more standard ADL/IADL practice task of picking up a cup. The cup was weighted to simulate the sensation of containing water (without the hardware safety risks that real water would present).
•An *experimental* tailored condition, in which the participant engaged in the less usual ADL/IADL practice task of picking up fake dog poop. This action was also framed with a prompt to the end user to try to scare or prank the caregiver or therapist by handing them this item or placing the item near them.

Each item was placed in one of two different locations:

•On a small table, about 2.8 ft (0.85 m) off of the ground.
•On the floor.

The items were assigned to locations in a balanced way across sessions, and the session always began with the tabletop item fetching task first.

### 7.3 Measurement

At the start and end of the evaluation overall (i.e., during the first and final sessions), we used the Box and Block Test (Mathiowetz et al., 1985) to assess the end user’s unilateral gross manual dexterity. We administered this test because we thought the experience of using the robot interface over multiple sessions could have a noticeable impact on hand dexterity. In the test, the participant had 60s to move as many of a set of 40 blocks from one holding box to another (initially empty) box.

For each task during each session, tasks were marked as completed in the live-transcribed field notes if the participant delivered the target item to the caregiver, therapist, or themselves. Meanwhile, the activities were considered incomplete if the participant did not fetch the item within 20 min of starting a given task condition. For tasks that were completed, we used a stopwatch to record the elapsed time between the activity start and end. A final behavioral measurement was the number of taps while using the interface.

Finally, we used a brief and verbally-administered set of subjective questions after each task (twice per session) to understand the child’s apparent experiences. This inventory was completed by the therapist since the child was unable to directly self-report this information, and the clinician had an established strong rapport with the participant to be able to gauge the child’s emotional responses. Using a a 7-pt Likert scale from “Strongly Disagree” to “Strongly Agree,” we asked the therapist to rate how engaged the child seemed to be, how much fun the child seemed to be having, and how happy the child seemed to be.

### 7.4 Procedure

**FIGURE 12:**
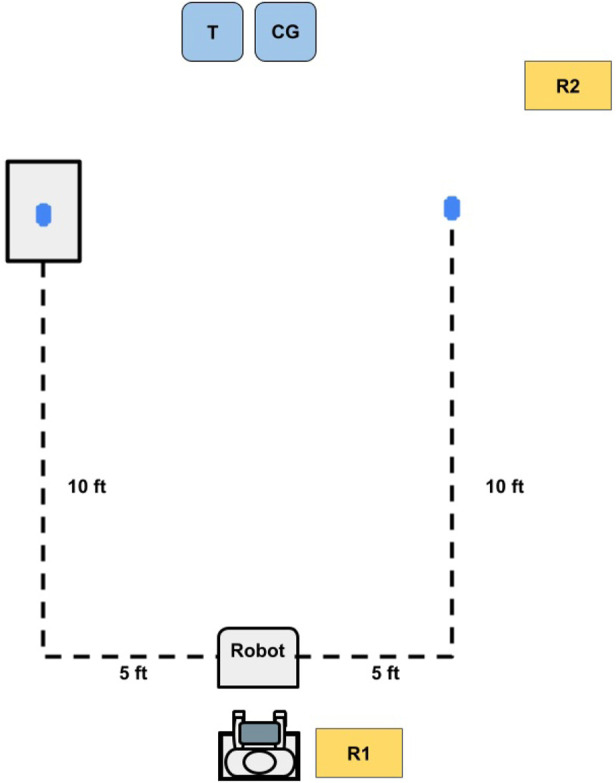
Top-down layout sketch of the evaluation environment. The participant operated the robot with the guidance of a researcher (R1) to pick up objects from one of two locations (represented by the blue ovals): the a table (left dot) and the floor (right dot). A second researcher (R2), recorded field notes during each session while the caregiver (CG) and therapist (T) were present in the environment for support as needed.

Before starting the first session of the evaluation, we administered the Box and Block Test. During each session, the robot and participant then took their positions at the starting locations shown in Figure 12. A researcher then placed one of the objects (i.e., cup or fake dog poop) in the appropriate location in the environment. After the object was placed in the environment, an iPad Pro running the interface via VNC viewer was given to the child and the child was prompted to collect the item. Following each condition, the robot was reset to its starting position and the therapist completed a post-condition survey. During the final session, the participant performed a closing Block and Box Test. Each session lasted about 30–45 min, with a total of four sessions in the full evaluation where the participant’s family received US$15 in compensation for completing each session.

### 7.5 Anticipated outcomes

During this evaluation we had three expectations regarding the effectiveness of the tailored activities, which were mediated using our custom robot interface. First, we expected that tailored activities would tend to lead to shorter completion times and higher engagement levels compared to standard ADL/IADL tasks, drawing on rehabilitation research that shows gamification to increase motivation and engagement (Adlakha et al., 2020). Second, we anticipated that using the robot would enhance hand dexterity through repetitive hand movements, as hinted by previous studies highlighting the benefits of such practices (Bütefisch et al., 1995). Lastly, we expected that participants would tend to report greater engagement, fun, and happiness during tailored activities compared to during standard tasks, based on observations from prototype testing where interaction with the robot elicitepd more joy and playful engagement.

### 7.6 Analysis

To evaluate how creating tailored scenarios can affect interactions in a robot-mediated pediatric OT setting, we assessed trending in the behavioral and subjective data, as further described below.

•Completion success data: The task completion information was logged based on the field notes.
•Completion time data: The completion time collected during each task was converted into seconds.
•User input data: The interaction data was collected from the robot’s logs, which included information about how many times the participant interacted with the interface. The UI input data rate was analyzed by computing the cumulative child inputs divided by the duration of each condition.
•Box and Block Test data: This assessment was scored by summing the total number of blocks moved during each test: the beginning and ending Box and Block Tests.
•Engagement and affect data: We used the therapist survey ratings to assess the apparent child levels of engagement, fun, and happiness.

Because child mood, alertness, and other factors varied from session to session, we visualize aggregate results for each condition (i.e., all baseline vs all experimental tailored interaction) to understand overall trends. We also discuss the session-wise trends in the text.

## 8 Follow-up evaluation results

The child participant was able to complete all tasks but two during the evaluation: the control condition from session one and the experimental condition from session 3. During these tasks, the participant appeared to be uninterested in fetching the item which lead to the therapist asking them if they wanted to move on and them agreeing. Each of these conditions lasted 20 min and were added as 20 min intervals in the completion time. The behavioral and subjective results for all task experiences, including the unsuccessful ones, are presented below.

### 8.1 Completion time results

**FIGURE 13:**
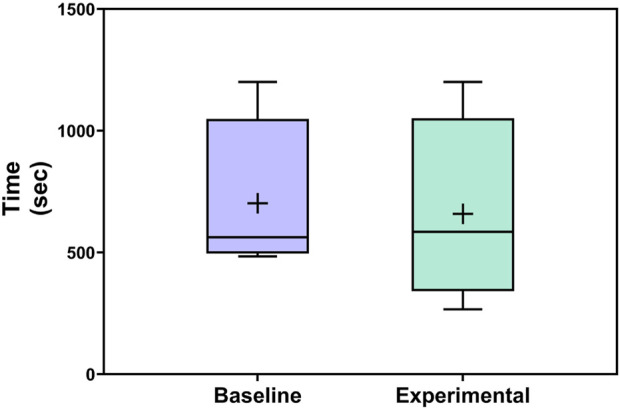
Completion time of each activity across conditions. Boxplots include boxes from the 25th to the 75th percentiles, center lines for medians, plus signs for means, and whiskers up to 1.5 times the interquartile range.

The completion time tended to be lower for the experimental condition compared with the control condition (where lower is better), as shown in Figure 13. The session-wise raw values varied, with at least one faster completion time occurring for each condition; however; at a session-wise level, different object locations were matched to each condition based on the counterbalancing. The aggregate view (which includes the same set of overall experiences for the two conditions) most clearly shows the overall trend in the data.

### 8.2 Input results

**FIGURE 14:**
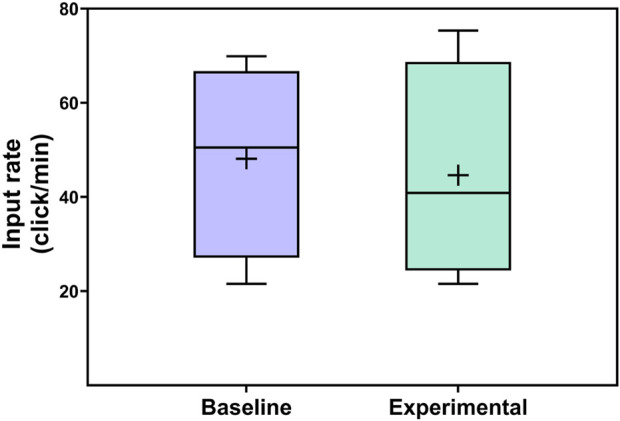
Distributions of child input rate across conditions.

The distribution of input data rate across conditions appears in Figure 14. The average input rate for the experimental condition tended to be lower compared to the control condition. As with the completion time data, the input rates at a session-wise level varied, with two lower input rates for each condition across the full study. Again, object location counterbalancing potentially contributed to these differences, and the overall trend may be most helpful for interpreting the data from the present work.

### 8.3 Box and block test results

The Box and Block scores showed an increase in gross manual dexterity over the course of the evaluation, with the participant collecting five more blocks (20 total blocks) in the final test compared to the initial test (15 total blocks).

### 8.4 Subjective results

**FIGURE 15:**
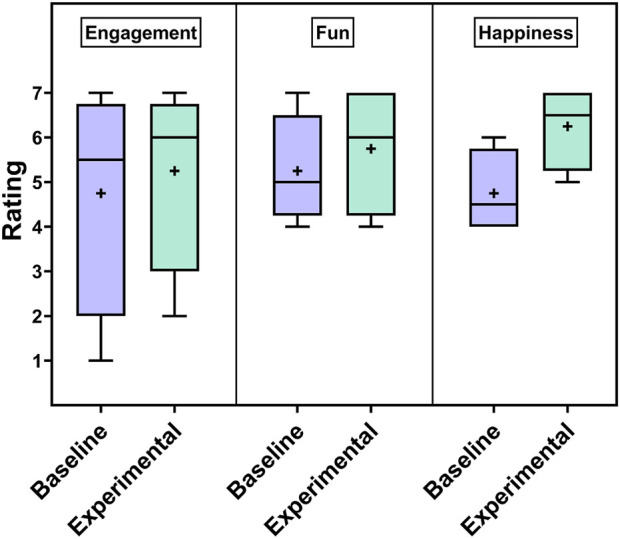
Ratings for perceived child engagement, fun, and happiness levels.

The results for perceived child engagement, fun, and happiness across conditions appear in Figure 15. The rated engagement, fun, and happiness levels tended to be higher for the experimental condition compared to the control condition. On an individual session-wise level, the experimental condition was rated as more engaging than the baseline in two cases, less engaging than the baseline in one, and equally engaging to the baseline in one. The session-wise breakdown for fun was similar, with two cases of more fun with the experimental condition, one of more fun with baseline, and one of equal fun for both conditions. For happiness, the ratings were higher for the experimental condition in three cases and equal to the baseline experience ratings in one case.

## 9 Overall discussion

In this work, we sough to evaluate if robot-mediated OT was viable for a child with cognitive and motor disabilities. To reach this goal we pursued and documented the process of designing and implementing a specialized user interface, in addition to evaluating the potential of this type of system when used in conjunction with tailored OT activities.

The result from our iterative interface design process showed that our efforts continually improved the ease and satisfaction of interface use for our child end user, in addition to confirming selected best practices from the assistive robotics field. For example, our early prototypes with multiple physical controls were overstimulating for the end user. This finding makes sense since the concept of complex control systems overwhelming end users is known (Chen et al., 2007), and this effect can be particularly salient for children with cognitive disabilities. Our later and more compact single-screen interface prototypes led to better overall performance by the end user. Generally, the level of success our end user experienced with the prototypes signals that young children with physical and cognitive disabilities can potentially use systems like the Stretch (or others even more adapted for child users) in pediatric OT experiences and beyond.

Based on observations during the design process, we zeroed in on a follow-up evaluation that allowed us to design and test a minorly improved robot interface, in addition to modulating the nature of the OT activities themselves. Specifically, the apparent enjoyment and motivation of our end user during playful tasks that involved minor mischief led us to wonder if lightly mischievous OT activities could enhance behavioral and subjective outcomes from therapy sessions. Generally, we saw faster task completion, fewer interface presses (a proxy for efficient robot control), and better ratings of engagement/affect for the experimental tailored activity condition (compared to the control condition). This trending is encouraging, and signals that lager follow-up experiments on implementing play (including the potential for mischief) and fun in pediatric OT would be well justified. Further, the results showed a trend of increased end user gross manual dexterity over the course of the experimental evaluation. Although it is difficult to isolate how much of this result is due to the end user’s existing therapy regimen vs our intervention in particular, the positive trend is encouraging. The preliminary success of the robot in a pediatric OT setting might extend to other applications, such as assistive use in grade school settings.

The *key strengths* of this effort include working directly with an end user with disabilities and his care network in the designing and testing prototypes, as well as evaluating tailored activities. This system of care included experts in the field of pediatric OT who provided feedback on all parts of the design process (e.g., creating prototypes, understanding the needs of the child participant) and also provided support throughout the implementation of tailored activities by suggesting tailored scenarios of interest for the end user. Without this network of caregivers and clinicians, it would not have been possible to conduct this work successfully. Among other reasons, this is because the network holds unique expertise on the particular end user we were engaging, and this network will in the end decide what solutions are usable and useful enough to merit longer-term adoption. An additional key strength is the positive trending of results across both major evaluations in the paper. We received continually better scores across the user interface design, and the follow-up evaluation showed positive trends for the tailored activities across the board. Due to (among other factors) variability in child mood between days and over the course of therapy sessions, this result is quite promising. At a broader level, we see this work as an example that could encourage more experimentation with robots in pediatric OT, toward the end goal of improving resources and outcomes for children with motor and cognitive disabilities.

The *limitations* included a small end user sample of just one participant in the design process and follow-up evaluation. Future work would need to consider experiences of other children undergoing OT, in addition to longer-term experiences with the considered type of assistive robot. Another limitation was the challenges that are associated when working with a young population and a population with disabilities, such as the fluctuation of interest and mood, and the specific health needs as well as legal considerations that one must take into account when working with a special population. For example, we were not able to record video footage of experiments that included a face view, to help protect the privacy of our end user. Further, for the subjective ratings, we relied on single-item scales and a therapist (not the child himself) to understand parts of the user experience. When possible, it would be best to collect this experiential information on validated scales and directly from the end user.

In *conclusion*, we implemented a design thinking process to design a robot control interface which was used by a child with cognitive and motor disabilities for pediatric OT. The system spaned a total of four evaluated prototypes and one final follow-up design. This last design was evaluated in a follow-up four-session experiment that also included tailored playful activities for potential use in robot-mediated OT. The results from the design process illustrated that simple one-screen interfaces with minimal controls tended to perform better than more complex systems. Our follow-up evaluation tended to show better behavioral and subjective results for tailored activities (compared to more traditional OT activities). Overall, this work shows the potential robots could have when implemented in pediatric OT settings, as well as how tailoring therapy activities for children can enhance outcomes. Insights from this work can inform a new sphere of assistive robotics efforts that can have an immense impact throughout life; the early cognitive and physical gains from the presented type of intervention can yield better outcomes during all life phases.

## Data Availability

The raw data supporting the conclusions of this article will be made available by the authors, without undue reservation.
